# The Perceptions of Trauma, Complaints, Somatization, and Coping Strategies among Syrian Refugees in Germany—A Qualitative Study of an At-Risk Population

**DOI:** 10.3390/ijerph17030693

**Published:** 2020-01-21

**Authors:** Ali Zbidat, Ekaterini Georgiadou, Andrea Borho, Yesim Erim, Eva Morawa

**Affiliations:** 1 Department of Psychosomatic Medicine and Psychotherapy, University Hospital of Erlangen, Friedrich-Alexander University Erlangen-Nürnberg (FAU), 91054 Erlangen, Germany; ali.zbidat@uk-erlangen.de (A.Z.); Ekaterini.Georgiadou@klinikum-nuernberg.de (E.G.); andrea.borho@uk-erlangen.de (A.B.); yesim.erim@uk-erlangen.de (Y.E.); 2 Department of Psychiatry and Psychotherapy, Paracelsus Medical University, 90419 Nürnberg, Germany

**Keywords:** refugees, Syria, trauma, coping, symptoms, somatization

## Abstract

**Background**: A high prevalence of mental distress, especially posttraumatic stress disorder, has been widely confirmed among refugees. In order to establish adequate interventions in psychotherapy, however, it must first be examined whether refugees have similar ideas and concepts of stress, trauma, and healing. This study, therefore, aimed to analyze the representations of trauma, self-reported complaints, indications of somatization, and coping strategies among a refugee population. **Methods**: Semi-structured interviews based on the Cultural Formulation Interview (CFI) of the Diagnostic and Statistical Manual of Mental Disorders (DSM-5) were conducted with Syrian refugees who have residence permission in Germany. The interviews were audio-recorded, transcribed, and analyzed according to the qualitative content analysis of Mayring. The foci of interest were determined on the basis of the predefined interview guideline, and inductive subcategories were extracted from the transcripts. **Results**: Sixteen refugees participated (50% women; mean age: 35.5 years, SD = 11.2; the mean duration of stay in Germany: 23.3 months, SD = 6.6). War experiences were the most frequently reported subjective perceptions of trauma. Frequently reported complaints included sleeping disturbance, cardiovascular symptoms, rumination, and pain. Among half of the participants, we found indications of somatization. We identified the following coping strategies: Activity, cognitive coping, social coping, religious coping, avoidance, and emotional coping. Conclusions: War-related traumatic events are the most common trauma perceptions among Syrian refugees. The self-reported complaints demonstrate somatoform, depressive, and posttraumatic symptoms. Syrian refugees should be screened for somatization, depression, and posttraumatic stress disorder and should receive targeted interventions that consider and support individual coping resources.

## 1. Introduction

According to estimates, the number of deaths in the war in Syria since 2011 totals about half a million; nearly six million people from the country were registered as refugees according to UNHCR (United Nations High Commissioner for Refugees) [1]. An enormous number of internally displaced persons is also assumed. In 2015, a hitherto unprecedented number of asylum seekers, a total of 1.1 million, immigrated to Germany. As many as 441,899 asylum applications were filed. Most initial applications (158,657) were from people from Syria. At 35.9%, Syrians represented one third of all the initial applications in 2015 in Germany [2].

Refugees are a special group of migrants, as their migration is not voluntary, unlike the case with migrant workers. Refugees flee their home country because of imminent or actual persecution due to, e.g., their political beliefs, religion, or nationality [3]. Legal refugee status can only be obtained as a decision of the host country in accordance with the Geneva Refugee Convention and the immigration laws of the country.

An involuntary migration from a region experiencing war or conflict is often associated with pre-, peri-, and post-migratory stressors. In their homeland, refugees flee from captivity, torture, violence, and murder, which, together with the often life-threatening escape itself, may represent severe and traumatic experiences. After migration, the unfamiliar environment of a new country, the acquisition of a new language and foreign culture in the host country, and the tense and cramped living conditions in their new accommodations place a burden on the mental health of refugees. In addition, in the host country, asylum seekers often struggle with isolation and stigmatization by the local population and face great uncertainty about their stay.

Refugees are exposed to more traumatic events, such as torture and acts of war or murder, than other populations before and during their escape from war zones and conflict regions, resulting in a higher vulnerability to the development of mental disorders, especially posttraumatic stress disorder (PTSD), depression, and somatization [4]. In a survey conducted among Syrian refugees in Turkey, PTSD was observed in one third of the participants [5]. Other recent studies with Syrian refugees also indicated an increased prevalence of PTSD [6] and depression [7]. Investigations into refugees since 1980 have been analyzed in a systematic review; refugees had high prevalence rates of depression (30.8%) and PTSD (30.6%) [8]. Another systematic review found significantly higher prevalence rates for mental illness among refugees than among migrant workers [9]. In a recent study, a prevalence rate of 40% for PTSD was observed among asylum seekers in Germany [10]. Another recent study of refugees in a German reception facility showed that the vast majority of participants suffer from PTSD, which is often comorbid with depression [11]. Another study in a reception facility detected one or more mental disorders (especially PTSD and depression) among refugees [12]. A recent survey described the symptoms of PTSD in 35.7% of asylum seekers living in collective housing and moderate to severe or severe depression among 58.9% [13].

Whether or not psychosocial distress leads to mental illness depends largely on coping strategies. In his stress model, Lazarus distinguished two possibilities of subjective coping: Problem-oriented and emotion-oriented coping. The former aims to re-evaluate the stress situation and to seek solutions or social support. Emotion-focused coping aims to reduce or control negative emotions, such as anxiety [14]. According to this stress model, previous studies have identified social support, avoidance, and problem solving (as appropriate) [15]; the early marriage of young girls and gender-based violence (as inappropriate mechanisms); and a shift in gender roles [16] as commonly used mechanisms among Syrian refugees. These refugees also establish relational-like relationships with their host families and access to the labor market as unskilled workers [17].

In German-speaking countries, there are hardly any studies on the highly heterogeneous refugee populations. Existing studies focus mainly on the prevalence rates of depression, PTSD, and generalized anxiety disorder [10,11,12,13]. There is, however, a major research deficit in the area of qualitative studies and coping strategies. As summarized above, previous studies indicate that Syrian refugees experience high mental distress due to their traumatic experiences. To develop appropriate psychotherapeutic interventions for them, we need better knowledge of the cultural representations of the traumatization and psychological coping among Syrians. Qualitative research on perceptions of trauma and coping with traumatic events is not available for Syrian refugees. The present survey attempts to fill this gap in the literature, so subsequent culturally sensitive therapy interventions can be developed based on this information.

This qualitative study examined participants’ understanding of trauma and their mental and physical complaints. Furthermore, coping strategies were investigated. 

## 2. Methods

This study was approved by the Ethics Committee of the Medical Faculty of the Friedrich-Alexander-University Erlangen-Nürnberg (FAU) (file reference: 74_17 B).

This study (Study II) was part of a larger study aimed at investigating the mental health of Syrian refugees, taking into account their living conditions in their home and host countries and their flight conditions (see Georgiadou et al.) [18]. All 518 Syrian refugees, who already held residence permissions, registered in the Erlangen Job center and were invited to participate in the questionnaire study. The inclusion criteria for the study were: Being a resident in Germany since 2014 (or shorter), having already reached the age of 18, and having statutory health insurance. Of the 518 Syrians mentioned above, 200 accepted the invitation to Study I (N = 200), which corresponds to 38.6% of the potential participants.

### 2.1. Interview

The data were collected by a semi-structured interview. This interview consisted of 17 questions, mostly based on the Cultural Formulation Interview (CFI) of Diagnostic and Statistical Manual of Mental Disorders 5 (DSM-5) [19]. These questions are shown in the Supplementary Material (File S1). As Mika et al. found, in a study of refugee disease concepts, some of the CFI’s original questions were too abstract and complicated to be understood correctly by the interviewees [20]. In their study, Mika et al. used the original English version of the CFI and determined various problem areas of understanding. The interviewees found it particularly difficult to move between spatial and temporal frames of reference. In order to prevent these misunderstandings, less abstract questions based on the CFI were used from the outset. There were also some additional self-constructed questions. The interview was semi-structured because an open dialogue with the interviewees was possible, and the interviewers inquired about interesting details to gain new insights beyond the interview questions. Based on the interview items, the following three questions were selected for the present study: (1) “How do you define a trauma, what is a trauma for you?”; (2) “What symptoms are you currently suffering from?”; and (3) “Which measures do you try to solve your complaints?”. These questions were selected because it was of particular interest what the participants themselves considered a trauma, regardless of the aspects of a scientific definition of trauma, which are frequently queried in the standardized questionnaires of quantitative studies. In addition, the complaints and individual coping strategies were also interesting from a psychotherapeutic point of view, as a suitable treatment can be developed and suggested based on personal perceptions and symptoms. The final version of the questions was then translated into Arabic in preparation for the interviews.

### 2.2. Recruitment

Out of the participants in the questionnaire study, 18 individuals, who had received positive screening for PTSD, depression, and/or generalized anxiety disorder and agreed to participate in the interview study, were randomly selected to participate with equal gender distribution in the present study. After an invitation by letter, the participants had the opportunity to choose a suitable date for the interview. Two persons moved to an unknown address, so the interviews were ultimately conducted with 16 respondents.

### 2.3. Participants

On average, the 16 participants were 35.5 years old (range = 21–55, SD = 11.2). Male and female refugees were equally represented. Ten people were married, and nine had at least one child. The average number of children was 1.8. The participants had been in Germany for an average of 23.3 months (range = 21–30 months, SD = 6.6). The escape journey of each refugee took an average of 2.4 weeks (range = 1–8 weeks). Most interviewees (10 participants) had an apartment with their family or lived alone. Thirteen of the respondents were Muslims, and two were Christians. One participant belonged to another denomination (see Table 1).

### 2.4. Procedure

During the 16 interview sessions that took place in November and December 2017 in Erlangen, the participants, the senior author (Yesim Erim), and a research associate (Ekaterini Georgiadou) were present. The first author of the article (Ali Zbidat), or a professional interpreter, was also present and acted as a cultural broker and interpreter. The discussions were recorded as an audio file with a recording device after the written consent of the participants. The interviews were conducted by Yesim Erim or Ekaterini Georgiadou, who asked the questions in German; then, the questions were read by Ali Zbidat or the professional interpreter in Arabic. The study participants received 25 € as compensation for their participation. The interviews lasted 49 min on average (range: 33 to 88 min).

### 2.5. Qualitative Method

The interviews were anonymized, transcribed, coded with the computer program Atlas.ti version 7.5.18 (Atlas.ti Scientific Software Development GmbH, Berlin, Germany), and evaluated using qualitative content analysis according to Mayring [21]. In a joint discussion among the authors (Yesim Erim, Eva Morawa, and Ali Zbidat), 16 foci of interest were first deductively formed based on the topics of the interview questions. For these definitions, anchor examples and coding rules were defined. Subcategories were independently and inductively extracted by two evaluators (Ali Zbidat and Andrea Borho) from the text material. Subsequently, in discussion rounds (Eva Morawa, Ali Zbidat, Andrea Borho), consensus versions of the subcategories were jointly assigned to four relevant foci of interest—traumatic experiences, health complaints, psychological coping (without the use of medical offers, since this forms part of another article), and references to somatization. The developed subcategories were thematically structured and grouped into main categories. In addition, the frequency of each subcategory was determined. Finally, the assignment of each subcategory was controlled.

## 3. Results

### 3.1. Events Described as Traumatic 

The majority of participants referred to war incidents that were experienced personally and/or witnessed as a trauma (Table 2). A drastic experience was, e.g., the (violent) deaths of close relatives or friends. One participant reported: "My mother was stabbed before me and my siblings. Then, we had to flee” (ID 11). Moreover, the sight of dead bodies was described as traumatizing, e.g., “Death, death, death, what you saw with your own eyes. Corpses of children, women, some burned in an unimaginable way" (ID 12). Detention and imprisonment were also defined as a trauma: “After they [Islamic State] arrested me, I was detained for 100 days under the threat of being killed every moment” (ID 15). Air strikes, explosions, life under the Islamic State, difficult living conditions (e.g., life without electricity and running water), and living in danger of death were found to be traumatic.

In addition to their war experiences, the refugees noted the deaths or illnesses of their relatives or friends as traumatic. Deaths or illnesses were often felt to be extremely distressing if they concerned their own children. The participants reported: "He was taken to a room to get an injection. They brought him back to me dead. They told me he had a cardiac arrest. If I remember this situation, I get very sad and worried” (ID 14) or “The photo speaks for itself. Even two pictures. A picture when he (the son) was in this condition (finger amputation), and one when he was dead” (ID 13).

Separation from family and the resulting feelings of guilt and helplessness were also considered traumatizing by some of the interviewed refugees. One participant described the stressful helplessness she felt towards her sisters who were left behind in Syria: “They are both old, ill, and alone. I have a bad conscience that I cannot help them, and they are in Syria, and I am here” (ID 2).

According to their subjective perceptions, the participants also reported traumatic events that only happened after fleeing to Germany. For example, a refugee reported the imprisonment of his brother: “Here in Germany, my other brother is in prison, which is also a great trauma for me” (ID 15). 

The participants also described physical or psychological violence in their families as possible traumatic experiences. This manifested itself, for example, as violence from one’s husband ("He hit me. One day the security service came in without knocking when they heard screams and noises; they witnessed the violence and called the police” (ID 11), beatings by one’s own mother (“My mother dealt harshly with us; we were beaten again and again" (ID 11)), or poor treatment by one’s father (“My father was a very bad person—worse than you can imagine. (...) He was not strict, it was more hate and humiliation” (ID 5)).

### 3.2. Complaints

The items in the interview guide on current complaints yielded a variety of answers. Very often sleep disorders, heart problems/palpitations, pondering, and pain were mentioned. An analysis of the responses to this question led us to form the following main categories: Mental and physical symptoms (see Table 3).

#### 3.2.1. Mental Symptoms

At the mental level, sleep disorders mainly manifested themselves as problems when falling asleep: “So far, I sleep very little. I wake up four, five, six, seven times at night. I had an appointment with the doctor to get sleeping pills” (ID 8) or “I promise you that I cannot remember a single day when I could sleep normally or fall asleep until two or three o’clock. My body is exhausted, and I have no strength left to keep my eyes open” (ID 16).

Many study participants reported emotional reactions. They often felt sad, depressed, or anxious. Some also showed increased excitability; they responded frequently and quickly with effervescent behavior and were very irritable. One participant reported: "My wife came to Germany for the first time five months ago and she noticed that something was wrong with me, e.g., I was quick-tempered and very angry from time to time in some situations" (ID 6). Next to homesickness and loneliness, the interviewees also experienced numbness: "When someone has so many experiences and losses in this war, he sometimes feels that he is neither sad nor satisfied, but between the two feelings" (ID 3).

Many interviewees complained about cognitive symptoms. They suffered from constant rumination and worrying. Their thoughts were circular, and their thinking could not be controlled or turned off. In this context, some of the participants suffered from a lack of concentration ("I have a lot of thoughts all day; my thoughts are always with my family in Syria; you try to concentrate, but there is no concentration" (ID 2)). One other participant reported forgetfulness. Also, flashbacks occurred, e.g., “I always see the same scenes from my childhood, as we were beaten by my parents” (ID 11). Occasionally, depression, mental fatigue or stress, social withdrawal, and tension were mentioned.

#### 3.2.2. Physical Symptoms

In addition to the psychological complaints described above, many physical symptoms were reported by the participants.

##### Cardiovascular Problems and Pain Symptoms

These problems often affected the cardiovascular system and were expressed as increased blood pressure, palpitations, or a sense of a stitch in the chest. "I went to the doctor. I asked him why I have these complaints and why my heart beats so fast. (...) He told me that I am well" (ID 10).

Several participants suffered from pain that occurred in different parts of the body. These included pain in the head, limb, abdomen, and back ("Last year I was there once or twice a month because of this pain, severe abdominal pain; even when I drank water, I got a stomachache" (ID 3)). Also, some felt physically exhausted and tired. Individual responses included loss of appetite, heartburn, loss of consciousness, trembling, and loss of control over one half of the face ("I had the feeling that I ... I do not know how to express it ... that I cannot control one half of my face."(ID 5)). Finally, thyroid problems were also reported.

##### Somatization

As can be seen from the previous examples, there was salient evidence of somatization. This evidence was identified in half of the interviewees. Somatization means that “the existing physical complaints imitate somatic diseases, without a sufficient organic reason present. Somatoform disorders can affect any organ system” [22] (p. 118).

First of all, there were cardiovascular and pulmonary symptoms, which were described as feelings of cardiac arrest, increased blood pressure, or chest pressure (see Table 4). A classic example of this phenomenon was found in the following description: "I have to go to the clinic sometimes, because I think a lot. I get breathless, tachycardia” (ID 10).

For this symptom group, the participants complained about severe pain in various other areas of the body, such as in the stomach or head. Particularly expressive was the following statement from a participant: "Yes, in the homeopathy center. Sometimes I go there because of my severe abdominal pain and migraines, especially after learning that my brother was killed” (ID 3).

One participant described hair loss, which, according to the doctor, was of psychological origin (ID 5). Another participant complained about her unfulfilled desire for children, which was attributed to mental health problems by her gynecologist: "She has urgently told me that I have no physical problems, but my mental health plays a role, so I cannot get pregnant" (ID 14).

### 3.3. Personal Coping

Interviewees were asked about the coping strategies they use to deal with their traumas and complaints. We classified individual strategies into the following six main categories: Activity, cognitive coping, social coping, religious coping, avoidance, and emotional coping (Table 5).

#### 3.3.1. Activity

Many refugees engaged in various activities that gave them relief: "When things are going bad, I usually go shopping with my husband" (ID 2) or "I do sports. I like to watch comedy programs and enjoy joking with friends" (ID 6).

#### 3.3.2. Cognitive Coping

In the context of cognitive coping, we identified several coping strategies, such as self-motivation, emotional control, future plans, self-talk, acceptance, and strength. "I always try to convince myself that I am stronger than the situation and can survive it" (ID 9).

#### 3.3.3. Social Coping

Reaching out to social contacts also had a positive influence on personal coping. For many participants, family cohesion and the well-being of the family were particularly relevant ("That my family is alright makes it a lot easier for me" said ID 1).

#### 3.3.4. Religious Coping

Other people reported religious coping. On the one hand, they accepted what happened and their situation as given by God; on the other hand, they used prayer as a coping strategy. One participant reported: "I prayed most of the time. That made me feel the most relieved" (ID 2).

#### 3.3.5. Avoidance

Some participants showed avoidance behavior. This was expressed, for example, by leaving Syria, fleeing from their problems, or avoiding hearings from home: "So my complaints do not get any worse, I avoid listening to the news and watching TV" (ID 8). 

#### 3.3.6. Emotional Coping

Emotional coping was shown by several participants in the form of quarreling and crying. "Whenever I cry, I feel better. No matter how many people talk and talk to me, the most important thing is to cry alone" (ID 14). 

## 4. Discussion

The aim of the present study was to explore the perceptions of trauma of Syrian refugees and their health complaints, as well as their coping strategies and possible indications of somatization.

### 4.1. Trauma

The DSM-5 defines trauma as the "exposure to actual or threatened death, serious injury or sexual violence (...)" [19] (p. 271). This confrontation occurs as a direct experience of a traumatic event (personally or in other persons), when a close family member or close friend has experienced a traumatic event, or as the repeated or extreme experience of incriminating details of a traumatic event. The present interviews showed that refugees designated events like violence and death as personal traumas, which corresponded to the aforementioned scientific definition. In contrast to quantitative surveys with questionnaires that query traumatic experiences based on this trauma definition, the refugees in the present study also named other representations of trauma that could be classified as onerous life circumstances, such as professional descent or separation from one’s family. Overall, these post-migratory stressors may be considered to have a greater influence on the manifestation and progression of mental illness than pre-migratory factors [23], as they present a situational psychological burden on migrants in their current life situations. Indeed, refugees experienced further traumatization in their host country, even after leaving their war-torn home and surviving their life-threatening escape. Maercker called posttraumatic stressors "the most influential (...) factors for the existence of chronic stress disorders (...)" [24] (p. 38).

### 4.2. Complaints

In the context of the complaints, many complaints were mentioned that coincide with the frequently asked symptoms of the questionnaire studies. Symptoms like increased excitability, sleep disturbances, or flashbacks after confronting a traumatic event speak in favor of PTSD. Furthermore, symptoms that may indicate depression, such as sadness, diminished one’s ability to concentrate. Insomnia or fatigue have also been reported frequently, which could be explained by the loss of one’s family, social fabric, and home [25].

The present evidence of somatization may be attributed to the traumatic events experienced by the participants. Kounou et al. also found that somatization among refugees may be a consequence of their trauma experienced, suggesting that trauma-centered therapies target not only the usual clinical picture of PTSD or depression but also possible evidence of somatoform disorder [26]. In Arabic culture, especially among women, somatoform disorder is often presented as so-called heart excitement, since there are few other ways to express emotional distress [27] (p. 76). This finding was supported by the research findings of female participants in our study who reported tachycardia without a physiological cause (e.g., ID 10). The reported somatoform disorders were also attributable to the most commonly diagnosed clinical conditions of PTSD, depression, and generalized anxiety disorder, and confirmed the findings of other studies with refugees on the prevalence rates of these disorders.

### 4.3. Coping Strategies

The finding in our study that the participants used a variety of coping strategies is in accordance with other studies on the coping behaviors of refugees. Gladden described, e.g., religious coping, re-interpretation of the situation, and social networks as coping strategies often used by different refugee populations [28]. The refugees performed dysfunctional behaviors (such as avoidance), as well as effective coping strategies (such as reaching out to social contacts, engaging in various activities or religious practices). 

The present results on the use of social contacts as a coping strategy confirm the great importance of family and are in line with Lazarus’ theory on coping mechanisms that describes, e.g., social support as a problem-oriented strategy of coping [14]. Family is very important in Arab culture; family members are often the first point of contact for problem management before seeking external advice or solutions to, e.g., psychological problems. The classical role distribution regards males as the dominant gender, which implies that men must be strong for their families. Although this behavior was described by male participants, some female interviewees also stated that they needed to be strong for their children and used this behavior as a coping strategy. We thus found a shift in traditional gender roles, which was also observed by El-Masri et al. in an assessment among Syrian and Palestinian refugees in Lebanon. They reported that refugees could no longer fulfill their traditional roles and explained their findings based on the devastating impact of the Syrian war on traditional gender roles and society [16]. 

Given that the majority of the sample studied is Muslim, the findings on religious practice as a coping strategy proved the important role of Islam and its influence on daily life. Muslims could interpret life events—including those of a traumatic nature—as an examination of their faith by God. Anyone who is faithful and firm in their belief in God will, from the Islamic point of view, accept and endure these trials patiently. Studies with Muslim refugees confirmed these conclusions; it is reported that Palestinian women used their faith to cope with their difficult daily life in a refugee camp [29]. Likewise, Sudanese refugees relied on religion as a coping mechanism, as religion provided both relief and a way to deal with their feelings of loneliness and depression [30]. The reported religious coping mechanisms (for example, prayer or general faith) were also determined to be effective tools in studies on Eritrean refugee women [31] and refugees in South Africa [32]; those refugees described prayer as a source of strength and hope. Prayers allowed the refugees to find comfort, guidance, and purpose in their difficult life situations and found that prayer could help them reduce the stress they experienced. Belonging to a religious community also provided further social support [33].

Avoidance behavior is part of the symptom cluster of PTSD, but here it was considered a coping strategy. In the present study, it was reported that contact with other refugees was avoided, to avoid being confronted again with traumatic memories. Our research findings differ from those of other studies, such as the work by Alzoubi et al., which stated that it is particularly important to build a functioning social network with other refugees [15]. Further clarification is needed to determine whether problem-oriented coping through social contacts should be preferred or rejected.

### 4.4. Strengths and Limitations

The strengths of the study are, firstly, that this research offers one of the first qualitative studies in the German-speaking world exclusively focused on Syrian refugees and their traumatic experiences and coping mechanisms. We thus gained new insights into refugees’ perceptions of trauma and coping strategies. Another strength of the study was that refugees with inadequate knowledge of German were also able to participate, as the interview in Arabic was conducted by a person of the same culture as the participants. This also counteracted a possible linguistic and cultural bias, so the findings were not distorted by linguistic and cultural misunderstandings.

At the same time, it cannot be ruled out that belonging to the same cultural group caused a certain level of inhibition among the participants towards the interviewer. To avoid possible condemnation or devaluation, the participants may deliberately have failed to mention practices that are considered "haram" (forbidden) in Islam, such as alcohol and drug use. Moreover, it is especially difficult for conservative Muslim women to talk to a stranger about shame-laced and taboo subjects, such as sexual violence or abuse. Although we did not find that the sincerity and openness of the participants was impaired during the interview, an interviewer of the same gender would have been more advantageous. Due to the exploratory and qualitative nature of the study, the results cannot be generalized to the entire community of Syrian refugees in Germany nor to the highly heterogeneous population of all refugees. Our results require replication in larger and more representative samples. Moreover, future studies should investigate other aspects of refugee resources.

## 5. Conclusions

As implications for the practical handling of refugees, and as a consequence of our results showing a high manifestations of distress, a regular screening for depression, PTSD, and somatization is recommended for refugees as soon as possible after their arrival in their host country. For the effective treatment of Syrian refugees, professionals should not only take classical traumatic events, such as death and torture, into consideration but should also consider post-migratory stressors. These stressors should be specifically discussed and taken into account. Early screening could identify persons who have clinical symptoms of distress and who are in need of treatment. In this way, one could prevent the chronification of their symptoms. Adequate treatment and mental health may be a prerequisite for the successful integration of refugees into society and the labor market or education system.

In order to be able to follow positive screening with effective treatment, a culturally sensitive provision of care for refugees is necessary. In psychotherapeutic work, it is important to gain insight into the beliefs of patients to avoid communication problems and misunderstandings and to strengthen their compliance with therapy. Refugees come from different cultural environments, and their cultural backgrounds and beliefs are essential determinants of their disease concepts and their handling of complaints. Therefore, sensitive recognition and management of cultural peculiarities is indispensable. This culturally specific care can be provided by specially trained personnel or by employees from a similar culture. For conservative Muslim women, as far as possible, attention should be paid to ensure the presence of female staff.

Since general practitioners are usually the first point of contact when people suffer from different symptoms, they should be sensitized to the main symptoms of refugees. For example, the most reported symptom in our study—sleeping disorder—could be an indication of PTSD or depression or both. Other physical complaints, like heart problems, without a sufficient organic reason could point to somatization.

The results of the present study indicate that Syrian refugees not only report their symptoms but also apply strategies to cope with their symptoms. Therapists should ask about existing coping strategies, give those strategies a positive connotation, and motivate their clients to use efficient coping mechanisms. We found that many participants in this study used activities like meeting friends or language acquisition and cognitive strategies, e.g., making plans, as coping strategies. Hence, refugees should be encouraged to use more of the strategies that they find helpful and also try other strategies.

## Figures and Tables

**Table 1:** Socio-demographic data.

ID	Gender	Age	Highest School Degree	School Attendance in Years	Learned Occupation in the Country of Origin	Last Practiced Profession in the Country of Origin	Marital Status	Religion	Children	Duration of Stay in Months	Flight Duration in Weeks
1	♀	36	n/a	14	n/a	n/a	married	Muslim	3	29	n/a
2	♀	55	n/a	0	n/a	n/a	married	Muslim	3	30	3
3	♀	31	high school	18	English teacher	n/a	single	Muslim	0	27	3
4	♂	23	n/a	12	secondary school	college student	single	Muslim	3	24	4
5	♂	23	high school	14	journalist	journalist	single	other	0	22	3
6	♂	29	university degree (economy)	8	manager	head of an electronic arcade	divorced	Muslim	0	21	3
7	♂	47	9th grade	9	tiler	tiler	married	Christian	5	25	n/a
8	♀	52	study (French)	17	language	n/a	married	Christian	1	25	n/a
9	♂	21	2 semesters of architecture	13	n/a	carpenter	single	Muslim	0	23	4
10	♀	40	primary school	7	tailor	tailor	married	Muslim	3	25	8
11	♀	24	n/a	9	n/a	n/a	divorced	Muslim	n/a	n/a	n/a
12	♀	45	7th grade	7	n/a	n/a	married	Muslim	3	25	1
13	♂	34	n/a	8	driver, seller	clothes seller	married	Muslim	0	24	8
14	♀	43	6th grade	8	hair stylist	hair stylist	married	Muslim	1	24	n/a
15	♂	42	computer science	7	electrician	electrician	married	Muslim	7	22	1
16	♂	23	secondary school	9	furniture shop	furniture shop	married	Muslim	0	26	n/a

n/a = not applicable.

**Table 2:** Events described as traumatic.

Subcategories	N *
War experiences (personally and/or as a witness)	13
Death/illness of relatives/friends	7
Subjective definitions (theoretical)	4
Physical/mental violence in the family	1
Depression	1
Separation from the family	1
Discrimination experience in Syria	1
Social/professional decline	1
Changeover from Syria to Germany	1
No place to return to	1

* Multiple answers possible.

**Table 3:** Complaints.

Main Categories	Subcategories	N *
Mental	Sleep disorders	12
	Emotional reactions	10
	Cognitive symptoms	10
	Mental exhaustion/stress	4
	Flashbacks	3
	Depression	2
	Social withdrawal	1
	Strain	1
	Exhaustion/fatigue	6
Physical	Pain symptoms	4
	Cardiovascular symptoms	3
	Dyspnea	2
	Problems with the thyroid gland	2
	Trembling/unrest	1
	Heartburn	1
	Pressure in the abdomen	1
	Nerve inflammation in the arm	1
	Loss of control over half the face	1
	Unconsciousness	1
	Mobility problems	1

* Multiple answers possible.

**Table 4:** Notes on somatization.

Subcategories	N *
Inconspicuous organic findings	4
Cardiovascular/pulmonary symptoms	3
Frequent visits to doctors	2
Respiratory symptoms	1
Strong pain	1
Hair loss	1
Loss of control over half the face	1
Unfulfilled desire for children	1
Frequent visits to the clinic due to shortness of breath and tachycardia through pondering	1
Illnesses because of constant fear	1

* Multiple answers possible.

**Table 5:** Personal coping.

Main Categories	Subcategories	N *
Activity	Music, singing	2
	Meeting friends, going out	2
	Being alone, quietness	2
	Distraction	2
	Language acquisition	2
	Laughing	1
	Smoking	1
	Shopping	1
	Sports	1
	Watching comedy programs	1
	Cooking	1
Cognitive coping	Making/having plans, goals	4
	Being strong/staying strong	3
	Self-motivation	2
	Emotion regulation	2
	Self-talk	2
Social coping	Conversations with family/friends	4
	Family reunion/family life	2
	Well-being of the family	2
	Reaching out to new social contacts	1
Religious coping	Praying	4
	Acceptance	2
Avoidance	Leaving the country	2
	Hearing no more battle noises	1
	Escape from problems	1
	Not hearing news	1
	No contacts with men	1
Emotional coping	Quarreling	2
	Crying	1

* Multiple answers possible.

## Supplementary Materials

The following are available online at https://www.mdpi.com/1660-4601/17/3/693/s1, File S1. Cultural Formulation Interview of the DSM-5 (CFI) (modified version).
