# Supplementary material for: The Perceptions of Trauma, Complaints, Somatization, and Coping Strategies among Syrian Refugees in Germany—A Qualitative Study of an At-Risk Population
Source: Int J Environ Res Public Health. 2020 Jan 21;17(3):693. doi: 10.3390/ijerph17030693 (PMC7037213; doi:10.3390/ijerph17030693)
Supplement: Supplementary file 1 [file ijerph-17-00693-s001.pdf]

File S1. Cultural Formulation Interview of the DSM-5 (CFI) (modified version).

1. What is a trauma / life crisis for you? Do you think that you have it or do you know someone with it?
2. Do you think you have mental distress? Do you know people who have a psychological burden from the trauma/life crisis? How do you explain these complaints? (if necessary, supplement: psychological stress can be e.g. sadness, sleep disorders, anxiety)
3. Does your problem occur frequently with your compatriots? Do you believe that your psychological complaints also occur in your home country?
4. Why do you think this happens to you or to others? In your opinion, what are the causes of these complaints?
5. Sometimes people describe their problems and complaints to their families, friends or others in their environment in different ways. If I was someone in your family, how would you describe your problem? Is there anything special that you would tell?
6. What do you think, what does / do your family / friends or other people think? Does it / do they also believe that the causes of the complaints are the same? And what does your family think will help you best with these complaints?
7. What worries you most about your complaints? What do you worry about the most/what do you fear the most?
8. Is there anything that can help you make your symptoms less severe, such as support from family, friends or others? What helps you? What can you do yourself? How can you help yourself? What do you do to help yourself? (if necessary: e.g. there are people who meet with friends, talk to family or try to alleviate their problems with cigarettes, alcohol, drugs etc.)
9. Is there anything that makes your problem worse, e.g. difficulties with money or family problems?
10. What have you done so far about your complaints? Did you seek help in the hospital or elsewhere? What have you done so far? e.g. other doctor, herbs, traditional healers. What does this mean to you? e.g. traditional healers. What do you think would help you best?
11. How do you find the doctors here compared to the doctors in your home country? Do you get along with the doctors here in Germany? Have you experienced anything special with the doctors in Germany?
12. Is the relationship to the doctor too authoritarian or not authoritarian enough? Do you consider yourself treated correctly or did you expect something different?
13. Does it make a difference whether the doctor is female or male?
14. Do you feel safe in Germany?
15. Can you imagine a life in Germany? How can you imagine the future in Germany?

Additional questions, if participants agree:

1. In your opinion, what are the differences between Germany and your home country regarding the relationship between men and women?
2. In your opinion, what are the differences between Germany and your home country regarding the relationship between young and old?
